# Paraspinal myosteatosis is associated with COPD: a cross-sectional MRI analysis from the population-based KORA cohort

**DOI:** 10.1186/s12931-025-03297-4

**Published:** 2025-06-14

**Authors:** Thierno D. Diallo, Stefan Karrasch, Matthias Jung, Annette Peters, Roberto Lorbeer, Christopher L. Schlett, Ricarda von Krüchten, Fabian Bamberg, Susanne Rospleszcz, Lena S. Kiefer

**Affiliations:** 1 Department of Diagnostic and Interventional Radiology, University Medical Center Freiburg, Faculty of Medicine, University of Freiburg, Hugstetter Strasse 55, 79106 Freiburg, Germany; 2 Institute and Clinic for Occupational, Social and Environmental Medicine, LMU University Hospital, LMU Munich, Munich, Germany; 3 Comprehensive Pneumology Center Munich (CPC-M), Member of the German Center for Lung Research (DZL), Munich, Germany; 4 Institute of Epidemiology, Helmholtz Zentrum München - German Research Center for Environmental Health (GmbH), Neuherberg, Germany; 5 Chair of Epidemiology, Institute for Medical Information Processing, Biometry and Epidemiology, Medical Faculty, Ludwig-Maximilians-Universität München, Munich, Germany; 6 Department of Radiology, LMU University Hospital, LMU Munich, Munich, Germany; 7 Department of Nuclear Medicine and Clinical Molecular Imaging, University Hospital Tuebingen, Eberhard Karls University of Tuebingen, Tuebingen, Germany; 8 Department of Diagnostic and Interventional Radiology, Eberhard Karls University of Tuebingen, Tuebingen, Germany

**Keywords:** Myosteatosis, Obstructive lung disease, Body composition, Magnetic resonance imaging, Imaging biomarker

## Abstract

**Background:**

Muscle dysfunction in chronic obstructive pulmonary disease (COPD) represents a significant extrapulmonary manifestation. Yet, the role of muscle fat infiltration (myosteatosis) in paraspinal muscles remains incompletely characterized. This study investigated whether paraspinal myosteatosis and its distribution patterns are associated with COPD and pulmonary function.

**Methods:**

Within the population-based KORA cohort, 214 participants underwent whole-body magnetic resonance imaging and pulmonary function testing. Paraspinal myosteatosis was quantified by chemical shift-encoded MRI at lumbar vertebra 3 (L3), from which proton density fat fraction (PDFF, in %) maps were derived. Intramyocellular (IMCL) and extramyocellular lipids (EMCL) were determined through voxel-based analysis using validated PDFF thresholds. COPD was defined spirometrically as FEV1/FVC below the lower limit of normal. Associations were examined using multivariable regression models adjusted for age, sex, smoking status, physical activity, and body mass index.

**Results:**

Among participants (mean age 58.5 ± 5.8 years, 56.1% male), 24 (11.2%) had spirometrically defined COPD. Participants with COPD showed higher paraspinal PDFF (19.9 ± 7.0% vs. 18.3 ± 7.6%) and lower IMCL/EMCL ratios (1.0 ± 0.4 vs. 1.2 ± 0.6) compared to those without COPD. After adjustment, higher PDFF was independently associated with increased odds of COPD (OR 1.69, 95% CI: 1.01–2.84, *p* = 0.046), while a higher IMCL to EMCL ratio showed protective associations (OR 0.49, 95% CI: 0.24-1.00, *p* = 0.050). Both total paraspinal PDFF and EMCL were negatively associated with pulmonary gas exchange capacity (TLCO/VA: β=-0.19, 95% CI: -0.35–0.04, *p* = 0.016 and β=-0.18, 95% CI: -0.33–0.03, *p* = 0.022, respectively). Conversely, higher IMCL/EMCL ratios were associated with better gas exchange (TLCO/VA: β = 0.15, 95% CI: 0.01–0.29, *p* = 0.031).

**Conclusions:**

This population-based study demonstrates that while increased total paraspinal muscle fat content is associated with higher COPD risk, its compartmental distribution reveals distinct patterns: A higher proportion of IMCL relative to EMCL shows protective associations, potentially reflecting preserved type I oxidative muscle fiber characteristics. These findings suggest that muscle fat distribution patterns may serve as imaging markers of metabolic adaptation in COPD, offering new perspectives for disease monitoring and therapeutic approaches.

**Supplementary Information:**

The online version contains supplementary material available at 10.1186/s12931-025-03297-4.

## Introduction

Chronic obstructive pulmonary disease (COPD) represents a significant health burden, and is considered a leading cause of morbidity and mortality worldwide [1]. It is characterized by chronic airflow obstruction leading to impaired pulmonary function, with profound effects on gas exchange and respiratory mechanics [2]. Beyond pulmonary manifestations, individuals with COPD often exhibit systemic effects, with skeletal muscle dysfunction being a key feature that substantially impacts functional status and quality of life [3]. Muscle dysfunction in COPD can manifest as decreased muscle strength, endurance, and mass [4]. While the loss of muscle mass (sarcopenia) has been extensively studied in respiratory diseases [5, 6], the role of muscle fat infiltration (myosteatosis) remains less well characterized. Myosteatosis represents qualitative muscle alterations that affect tissue architecture and metabolic function. It can manifest independently of sarcopenia and may even precede muscle mass loss [7]. In skeletal muscle, fat can accumulate as intramyocellular lipids (IMCL), stored as lipid droplets within muscle fibers serving as physiological energy substrates during oxidative metabolism particularly in type I fibers, and as extramyocellular lipids (EMCL). EMCL represent ectopic fat depots stored in adipocytes, either between muscle fibers within individual muscles (intramuscular), between muscles and their fascia or between muscle groups (both intermuscular) [8, 9]. Intermuscular adipose tissue, acts as an endocrine organ producing inflammatory mediators that can directly affect skeletal muscle metabolism [10, 11]. Consequently, not only the total amount of muscle fat but also its distribution between intra- and extramyocellular compartments may provide insights into muscle metabolism and function.

Chemical shift encoded magnetic resonance imaging (MRI) provides a non-invasive method for detailed assessment of muscle mass and quantitative evaluation of myosteatosis while allowing the differentiation of IMCL and EMCL compartments [12]. Several studies have established myosteatosis as an important prognostic marker across various conditions and as a strong predictor of all-cause mortality in both individuals with metabolic disorders and asymptomatic adults [7, 13–15].

Despite evidence linking muscle dysfunction with COPD, the associations between MRI-derived muscle parameters, especially myosteatosis, and COPD in the general population remain underexplored. Therefore, we investigated associations between paraspinal myosteatosis and its distribution in relation to spirometrically defined COPD in a sample of adults from a population-based cohort. We hypothesized that increased muscle fat content would be associated with increased prevalence of COPD, and that IMCL and EMCL would demonstrate different associations with COPD and related respiratory parameters.

## Materials and methods

### Study sample

We used data from the cross-sectional MRI substudy within the “Cooperative Health Research in the Region of Augsburg” (KORA) cohorts [16]. The MRI examination was done within the second follow-up KORA-FF4 (*N* = 2279, enrolled in 2013/2014) of the original population-based KORA-S4 survey (*N* = 4261, enrolled in 1999/2001). Of the KORA-FF4 cohort, *N* = 400 participants underwent whole-body MRI within 33 days (interquartile range: 24–45 days) of their examination at the study center. Inclusion criteria were no contraindications to MRI (claustrophobia, allergy to contrast agent, permanent metal parts in the body), age < 74 years, being in generally good health sufficient for the one-hour whole-body imaging procedure, and no prevalent cardiovascular disease (stroke, myocardial infarction, revascularization) [16]. Additionally, *N* = 1010 participants of the KORA-FF4 cohort in the age range 48–68 years underwent pulmonary function testing [17]. The overlap of these two samples constitutes the analytical sample for the current analysis. The Ethics committee of the Bavarian Chamber of Physicians approved the general KORA cohort studies. The Ethics Committee of the Ludwig-Maximilians University Munich (Munich, Germany) additionally approved the whole-body MRI substudy (No. 498 − 12). The study was carried out in accordance with the Declaration of Helsinki and obtained written informed consent from all participants. Clinical trial number: not applicable.

### MRI protocol and muscle segmentation

**Fig. 1:**
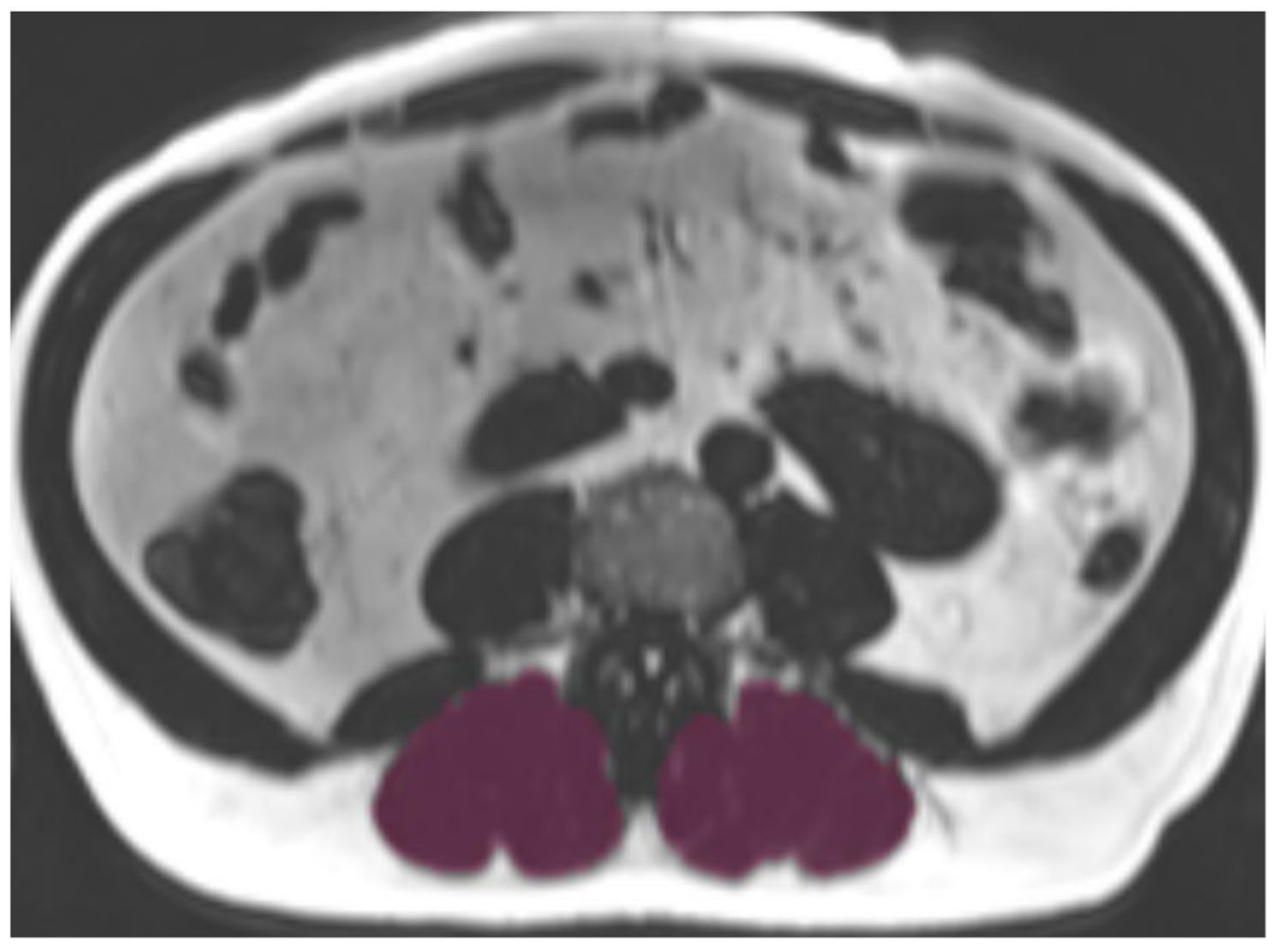
Representative axial T2*-corrected multi-echo Dixon MRI slice (fat contrast) at level L3 with superimposed paraspinal muscle segmentation (purple). Manual segmentation was performed bilaterally using standardized anatomical landmarks **Abbreviations**: MRI: magnetic resonance imaging; L3: 3rd lumbar vertebra

MRI examinations were performed using a 3 Tesla Magnetom Skyra scanner (Siemens Healthineers, Erlangen, Germany) with combined 18-channel body surface and spine matrix coils. The complete imaging protocol and technical specificities have been described in detail previously [16]. For muscle analysis, T2*-corrected multi-echo 3D-gradient-echo Dixon sequences of the upper abdomen were acquired during a single breath-hold (slice thickness 4 mm, voxel size 1.8 × 1.8 mm, field-of-view 393 × 450 mm, matrix 256 × 179, TR 8.90ms, TEs 1.23, 2.46, 3.69, 4.92, 6.15, 7.38ms, flip angle 4°). Accurate transverse slice positioning at L3 vertebral level was confirmed using coronal two-point Dixon gradient-echo sequences (TR 4.06ms, TE 1.26 and 2.49ms, flip angle 9°, slice thickness 1.7 mm, isotropic in-plane resolution 1.7 mm) with cross-reference. Paraspinal muscles were manually segmented at the level of the third lumbar vertebra by two observers blinded to clinical data and COPD status using standardized anatomical landmarks (MITK V2015.5.2, German Cancer Research Center, Heidelberg, Germany). Skeletal muscle area (mm^2^) was defined as the sum of the left and right areas of the paraspinal muscles (Fig. 1).

### Myosteatosis quantification

Total myosteatosis was determined as proton density fat fraction (PDFF, in %). PDFF maps were calculated from the original data using the manufacturer’s software (MR Liverlab, Version VD13, Siemens Healthineers, Cary, USA). Mean PDFF was calculated by averaging measurements from right and left muscle compartments, including IMCL, intramuscular and subfascial intermuscular EMCL, while excluding surrounding extrafascial intermuscular EMCL. The reproducibility of this segmentation approach was previously validated in a methodological study using the same protocol, demonstrating excellent reliability with intraclass correlation coefficients of 0.98 and 0.99 for inter- and intra-observer agreement of PDFF measurements, and 0.97 and 0.98 for muscle area measurements [18].

For differentiation of fat compartments, IMCL were quantified by postprocessing the segmented muscle compartments using a semi-automated algorithm (Matlab_R2017a; MathWorks, MA, USA). This algorithm applies an established threshold based on the understanding that myocytes with high intramyocellular lipid content demonstrate values ≤ 20%, while higher values indicate the presence of extramyocellular adipose tissue [12]. This 20% PDFF threshold follows the physiological rationale, where values below 20% reflect small intracellular lipid droplets and above 20% larger adipocyte aggregates [19]. Accordingly, voxels with PDFF values ≤ 20% were classified as containing IMCL, while EMCL content was calculated as the difference between total paraspinal myosteatosis and IMCL, comprising predominantly extramyocellular intramuscular fatty septa and subfascial intermuscular adipose tissue [18].

### Pulmonary function testing

As reported previously [20], three categories of pulmonary function indices were analyzed: (1) Spirometric indices including forced vital capacity (FVC), forced expiratory volume in 1 s (FEV1), FEV1/FVC, and forced expiratory flow between 25% and 75% of FVC (FEF25–75); (2) lung volumes derived from single-breath measurement of pulmonary gas exchange, which include residual volume (RV), functional residual capacity (FRC), total lung capacity (TLC), and alveolar volume (VA); (3) indices of pulmonary gas exchange, which include transfer factor of the lung for carbon monoxide (TLCO) and the transfer coefficient TLCO/VA. Standard spirometry without bronchodilation and single-breath measurement of TLCO were performed in line with German as well as American Thoracic Society (ATS)/European Respiratory Society (ERS) recommendations in upright sitting position using a pneumotachograph-type spirometer (MasterScope PC and MasterScreeen PFT, CareFusion, Höchberg, Germany) [21]. At least three and up to eight spirometric maneuvers and up to five single-breath TLCO maneuvers were performed to obtain a minimum of two acceptable and reproducible measurements. COPD was defined as FEV1/FVC below the Lower Limit of Normal (LLN) based on American Thoracic Society and European Respiratory Society recommendations using Global Lung Initiative reference values that are based on age, sex, height and ethnicity [22, 23].

### Clinical covariates

All participants underwent standardized interviews and physical examinations at the study center. Anthropometric measurements, including weight, height, and body mass index (BMI), were obtained according to standardized protocols. Blood pressure measurements were performed to assess hypertension, defined as systolic blood pressure ≥ 140 mmHg, diastolic blood pressure ≥ 90 mmHg, or current antihypertensive medication. Laboratory analysis of fasting blood samples included the determination of blood lipid parameters (HDL, LDL, and triglycerides) and inflammatory markers (hsCRP) by standard laboratory measures. Smoking behavior was assessed by self-report and classified into never, former and current smoker. Study participants were classified as physically active (regular physical activity ≥ 2 h/week or approximately 1 h/week) or physically inactive (irregular physical activity < 1 h/week, almost no or no physical activity), based on self-report.

### Statistical methods

Participants’ baseline characteristics for the whole sample and stratified by COPD status are presented as mean and standard deviation or median with interquartile range for continuous data, and counts and percentages for categorical data. Differences between groups were quantified by t-test or Wilcoxon-test, and χ^2^ test or Fisher’s exact test for groups with *n* < 5, respectively.

Univariate correlations between MRI-derived skeletal muscle parameters and pulmonary function were quantified by Spearman’s correlation coefficient.

To evaluate the association between MRI-derived skeletal muscle parameters as exposure and pulmonary function parameters as an outcome, adjusted linear and logistic regression models were applied. Models were adjusted for 1) age and sex, 2) age, sex, smoking, diabetes, physical activity (yes/no), and BMI, and 3) Model 2 plus hsCRP. All exposures and continuous outcomes were centered and scaled (minus mean and divided by standard deviation) before modelling. Results are given as beta coefficients or Odds Ratios (OR) with respective 95% confidence intervals.

R version 4.4.1 was used for statistical analysis. *P*-values < 0.05 were considered to denote statistical significance of associations, without adjustment for multiple testing.

## Results

### Study sample

**Fig. 2:**
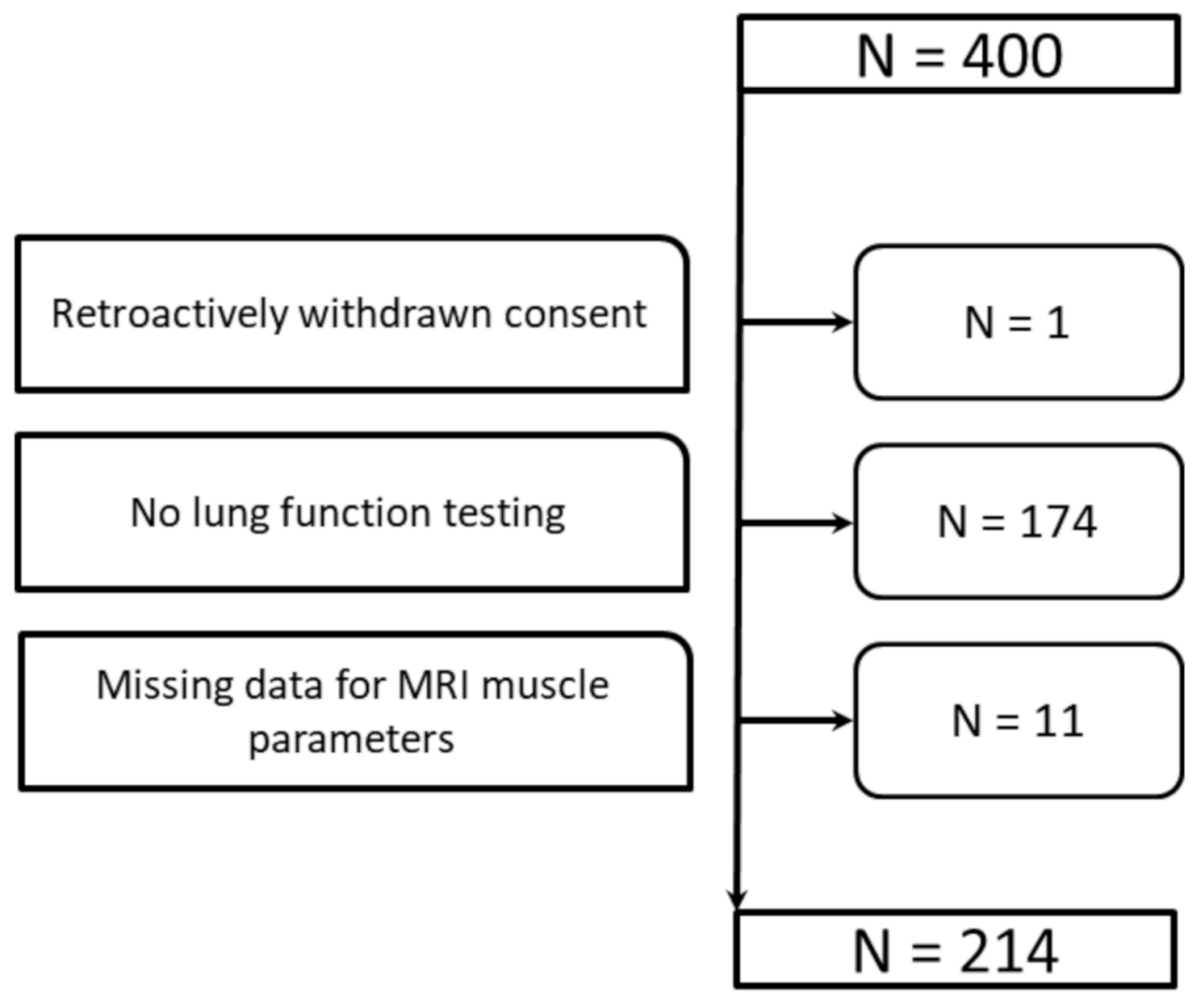
Participant Flowchart Flowchart showing the inclusion and exclusion process for study participants. Total number of assessed participants (*n* = 400) included (*n* = 214), and excluded individuals with reasons for exclusion

Of the *N* = 400 individuals originally enrolled in KORA-MRI, one retroactively withdrew consent for data usage, *n* = 174 did not undergo pulmonary function testing, and *n* = 11 had no data on skeletal muscle area and PDFF from MRI. Therefore, the final analytical sample comprised *N* = 214 participants (Fig. 2), with varying sample sizes for some variables (Table 1).

**Table 1:** Participants’ clinical characteristics, muscle composition, and pulmonary function

	All	No COPD (LLN)	COPD (LLN)	*p*-value
	*N* = 214	*N* = 190	*N* = 24	
**Demographics**				
Age, years	58.5 ± 5.8	58.6 ± 5.6	57.2 ± 6.7	0.276
Men	120 (56.1%)	104 (54.7%)	16 (66.7%)	0.373
**Body Composition**				
Height, cm	171.0 ± 10.0	170.8 ± 9.8	172.9 ± 11.8	0.685
Weight, kg	83.8 ± 15.9	83.9 ± 15.8	82.5 ± 16.5	0.330
BMI, kg/m^2^	28.6 ± 4.7	28.7 ± 4.7	27.6 ± 5.1	0.290
BMI ≥ 30 kg/m^2^	69 (32.2%)	63 (33.2%)	6 (25.0%)	0.566
Waist circumference, cm	100.2 ± 13.4	100.3 ± 13.2	99.4 ± 15.1	0.739
Body Surface Area, m^2^	2.0 ± 0.2	2.0 ± 0.2	2.0 ± 0.2	0.948
**Lipid Profile**				
Total Cholesterol, mg/dL	222.4 ± 37.2	222.5 ± 36.3	221.6 ± 44.4	0.918
Triglycerides, mg/dL (median [Q1, Q3])	113.0 [83.2, 163.6]	112.5 [84.1, 154.5]	124.3 [66.5, 198.2]	0.664
HDL, mg/dL	62.2 ± 17.7	62.7 ± 17.8	58.4 ± 16.6	0.267
LDL, mg/dL	142.9 ± 34.4	143.0 ± 33.8	142.0 ± 39.5	0.898
Lipid lowering medication	27 (12.6%)	24 (12.6%)	3 (12.5%)	0.332
**Glycemia**				
Status				0.730
normogylcemia	121 (56.5%)	108 (56.8%)	13 (54.2%)	
prediabetes	60 (28.0%)	54 (28.4%)	6 (25.0%)	
diabetes	33 (15.4%)	28 (14.7%)	5 (20.8%)	
Fasting glucose, mg/dL	106.3 ± 21.7	106.2 ± 22.3	106.7 ± 16.7	0.918
HbA1c, %	5.6 ± 0.6	5.6 ± 0.7	5.7 ± 0.4	0.748
Glucose lowering medication	19 (8.9%)	17 (8.9%)	2 (8.3%)	0.773
**Blood Pressure**				
Systolic blood pressure, mmHg	122.1 ± 16.0	121.9 ± 15.9	123.6 ± 17.1	0.640
Diastolic blood pressure, mmHg	76.8 ± 9.7	77.0 ± 9.9	74.6 ± 7.8	0.254
Hypertension	85 (39.7%)	76 (40.0%)	9 (37.5%)	0.988
Antihypertensive medication	64 (29.9%)	59 (31.1%)	5 (20.8%)	0.427
**Lifestyle Factors**				
Physical Activity				0.841
regularly 2 h/w	59 (27.6%)	52 (27.4%)	7 (29.2%)	
regularly 1 h/w	69 (32.2%)	62 (32.6%)	7 (29.2%)	
sporadically	31 (14.5%)	27 (14.2%)	4 (16.7%)	
inactive	55 (25.7%)	49 (25.8%)	6 (25.0%)	
Smoking				0.485
never-smoker	82 (38.3%)	75 (39.5%)	7 (29.2%)	
ex-smoker	87 (40.7%)	77 (40.5%)	10 (41.7%)	
smoker	45 (21.0%)	38 (20.0%)	7 (29.2%)	
**Inflammation**				
hsCRP, mg/L (*N* = 211)	1.2 [0.7, 2.8]	1.2 [0.7, 2.6]	1.4 [0.8, 5.7]	
**Lung Volume**, **MRI (*N* = 213)**				
total, L	4.0 ± 1.1	3.9 ± 1.0	4.8 ± 1.5	< 0.001
left, L	1.8 ± 0.6	1.8 ± 0.5	2.2 ± 0.7	< 0.001
right, L	2.2 ± 0.6	2.1 ± 0.6	2.6 ± 0.8	< 0.001
**Skeletal Muscle**, **MRI**				
Area, mm^2^	4945.9 ± 1124.4	4949.1 ± 1131.3	4920.6 ± 1091.5	0.907
Myosteatosis, PDFF %	18.5 ± 7.6	18.3 ± 7.6	19.9 ± 7.0	0.336
IMCL, % (*N* = 206)	8.3 ± 2.8	8.4 ± 2.9	7.9 ± 1.7	0.466
IMCL, %, median [Q1, Q3] (*N* = 206)	7.8 [6.4, 9.7]	7.8 [6.3, 9.8]	7.7 [6.8, 8.4]	
EMCL, % (*N* = 206)	9.0 ± 5.7	9.0 ± 5.7	9.3 ± 5.3	0.800
EMCL, %, median [Q1, Q3] (*N* = 206)	8.0 [4.7, 11.2]	8.0 [4.6, 11.5]	8.5 [6.4, 9.6]	
IMCL/EMCL, (*N* = 206)	1.2 ± 0.6	1.2 ± 0.6	1.0 ± 0.4	0.135
IMCL/EMCL, median [Q1, Q3] (*N* = 206)	1.0 [0.8, 1.4]	1.0 [0.8, 1.4]	0.9 [0.8, 1.2]	
**Pulmonary function**				
RV, L (*N* = 206)	2.13 ± 0.40	2.09 ± 0.37	2.44 ± 0.48	< 0.001
FRC, L (*N* = 204)	2.86 ± 0.68	2.82 ± 0.66	3.16 ± 0.75	0.021
VA, L (*N* = 206)	6.02 ± 1.21	5.98 ± 1.20	6.33 ± 1.31	0.176
TLC, L (*N* = 206)	6.20 ± 1.22	6.16 ± 1.21	6.51 ± 1.32	0.184
FVC, L	4.12 ± 1.03	4.10 ± 1.03	4.28 ± 1.06	0.424
FEV_1_/FVC	74.92 ± 7.67	76.84 ± 5.23	59.71 ± 6.97	< 0.001
FEF_25 − 75_	2.45 ± 0.93	2.61 ± 0.86	1.21 ± 0.50	< 0.001
TLCO, mmol/min/kPa (*N* = 206)	8.40 ± 1.99	8.42 ± 1.96	8.24 ± 2.22	0.686
TLCO/VA, mmol/min/kPa/L (*N* = 206)	1.40 ± 0.19	1.41 ± 0.19	1.30 ± 0.19	0.006

Values are presented as arithmetic means and standard deviations (or otherwise indicated) with *p*-values from t-test (or Mann-Whitney-U test), or counts and percentages with *p*-values from Χ^2^-test or Fisher’s exact test

**Abbreviations**: BMI: body mass index; EMCL: extramyocellular lipids; FEF_25 − 75_: forced expiratory flow between 25% and 75% of FVC; FEV_1_/FVC: Tiffenau-Index; FRC: functional residual capacity; FVC: forced vital capacity; HbA1c: glycated hemoglobin; HDL: high density lipoprotein; hsCRP: high sensitivity C-reactive protein; IMCL: intramyocellular lipids; LDL: low density lipoprotein; MRI: magnetic resonance imaging; PDFF: proton density fat fraction; RV: residual volume; TLC: total lung capacity; TLCO: transfer factor of the lung for carbon monoxide; VA: alveolar volume

Individuals of the original KORA-MRI sample that were excluded from the current analysis were, on average, younger and had lower cardiometabolic risk factor levels (Supplementary Table 1). Individuals in the sample were 58.5 ± 5.8 years old, 56.1% were men, and 21% were active smokers (Table 1). COPD, as defined by LLN, was present in 24 (11.2%) of individuals. Those with COPD had nominally higher PDFF and EMCL but nominally lower IMCL and IMCL/EMCL ratios (Table 1).

**Fig. 3:**
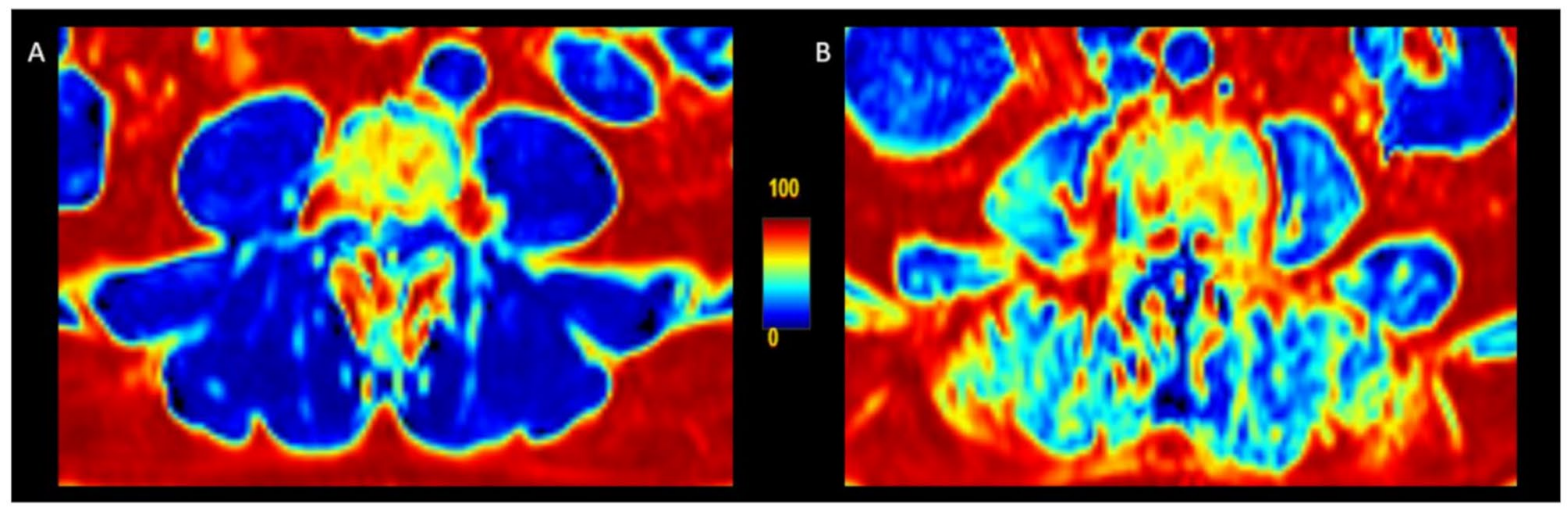
Proton density fat fraction (PDFF) maps of paraspinal muscles at level L3 showing (**A**) low muscle fat content in an individual without COPD (PDFF 4.5%, FEV_1_/FVC 70.8) and (**B**) high muscle fat content in an individual with COPD (PDFF 38.8%, FEV_1_/FVC 54.4). Color scale indicates PDFF values from 0% (blue) to 100% (red) **Abbreviations**: PDFF: proton density fat fraction; L3: 3rd lumbar vertebra; COPD: chronic obstructive pulmonary disease; FEV_1_/FVC: Tiffenau-Index

Exemplary PDFF maps are shown in Fig. 3.

### Correlation between MRI-derived skeletal muscle parameters and pulmonary function

In univariate analysis, skeletal muscle and pulmonary parameters were correlated to different degrees, ranging from a Spearman correlation coefficient of -0.51 between PDFF and FVC, to a correlation coefficient of 0.59 between skeletal muscle area and TLCO (Supplementary Fig. 1). We note that particularly the IMCL/EMCL ratio showed stronger correlations with pulmonary parameters in individuals with COPD compared to individuals without COPD (Supplementary Fig. 1).

### Association of MRI-derived skeletal muscle parameters with pulmonary function and COPD

**Fig. 4:**
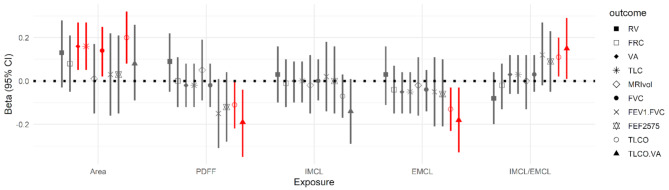
Association between muscle parameters and outcomes of lung volumes, obstruction, and gas exchange Presented are beta coefficients and corresponding 95%CIs (on the y-axis) from a linear regression model with exposure muscle parameters (on the x-axis), as derived by MRI, and outcomes of pulmonary volumes, obstruction and gas exchange. Results with *p* < 0.05 are displayed in red. Presented are results from the model with full adjustment: Adjusted for age, sex, smoking, diabetes, physical activity (yes/no), and BMI. All exposures and outcomes were standardized before modelling, indicating change in the standard deviation of the pulmonary function outcome by one standard deviation change in the skeletal muscle exposure **Abbreviations**: BMI: body mass index; EMCL: extramyocellular lipids; FEF_25 − 75_: forced expiratory flow between 25% and 75% of FVC; FEV_1_/FVC: Tiffenau-Index; FRC: functional residual capacity; FVC: forced vital capacity; IMCL: intramyocellular lipids; MRIvol: MRI (magnetic resonance imaging) derived lung volumes; PDFF: proton density fat fraction; RV: residual volume; TLC: total lung capacity; TLCO: transfer factor of the lung for carbon monoxide; VA: alveolar volume

After full adjustment, there were physiologically plausible associations between MRI-derived muscle area with VA (beta = 0.16, 95%CI: [0.05, 0.27], *p* = 0.005), TLC (beta = 0.16, 95%CI: [0.05, 0.27], *p* = 0.004), FVC (beta = 0.14, 95%CI: [0.02, 0.25], *p* = 0.017) and TLCO (beta = 0.20, 95%CI: [0.08, 0.32], *p* = 0.001, Fig. 4 and Supplementary Tables 2–4).

However, there was no association between skeletal muscle areas with the presence of COPD (Table 2). PDFF was not associated with any parameter of pulmonary volume (Fig. 4, Supplementary Table 2), but an increase PDFF was tentatively associated with a decrease in FEV_1_/FEV (beta=-0.15, 95%CI: [-0.31, 0.01], *p* = 0.07, Fig. 4 and Supplementary Table 3), as well with a decrease in TLCO (beta=-0.11, 95%CI: [-0.22, 0.00], *p* = 0.043) and TLCO/VA (beta=-0.19, 95%CI: [-0.35, -0.04], *p* = 0.016, Fig. 4 and Supplementary Table 4). An increase in one standard deviation of PDFF was associated of a 1.69 fold increase in risk for COPD (*p* = 0.046, Table 2).

**Table 2:** Association between MRI-derived skeletal muscle parameters and presence of obstructive lung disease

Exposure	Adjustment	Outcome COPD		
		OR	95%-CI	*p*-value
Area (mm^2^)	Age + sex	0.71	[0.39, 1.27]	0.250
	Full	0.75	[0.40, 1.41]	0.368
	Full + hsCRP	0.87	[0.47, 1.63]	0.669
PDFF (%)	Age + sex	1.62	[1.01, 2.58]	0.044
	Full	1.82	[1.10, 3.02]	0.020
	Full + hsCRP	1.69	[1.01, 2.84]	0.046
IMCL (%)	Age + sex	0.97	[0.59, 1.59]	0.889
	Full	1.02	[0.61, 1.73]	0.926
	Full + hsCRP	1.05	[0.60, 1.86]	0.861
EMCL (%)	Age + sex	1.31	[0.82, 2.09]	0.259
	Full	1.44	[0.87, 2.38]	0.154
	Full + hsCRP	1.35	[0.81, 2.26]	0.252
IMCL/EMCL	Age + sex	0.52	[0.28, 0.98]	0.043
	Full	0.46	[0.23, 0.93]	0.030
	Full + hsCRP	0.49	[0.24, 1.00]	0.050

Results from a logistic regression model with exposure skeletal muscle parameters, as derived by MRI, and outcome COPD defined as FEV1/FVC below the lower limits of normal. Full adjustment: Adjusted for age, sex, smoking, diabetes, physical activity (yes/no), and BMI. All exposures were standardized before modeling, and Odds Ratios (OR) are given per change in standard deviation

**Abbreviations**: BMI: body mass index; EMCL: extramyocellular lipids; FEV_1_/FVC: Tiffenau-Index; IMCL: intramyocellular lipids; PDFF: proton density fat fraction

There were no associations of IMCL with pulmonary volumes or obstruction parameters (Fig. 4 and Supplementary Tables 2 and 3), but increased IMCL was tentatively associated with decreased TLCO/VA (beta=-0.14, 95%CI: [-0.29, 0.01], *p* = 0.074, Fig. 4 and Supplementary Table 4). Changes in IMCL were not associated with the presence of COPD (Table 2). EMCL showed a similar trend of associations, with a more pronounced association for the decrease in TLCO (beta=-0.13, 95%CI: [-0.23, -0.03], *p* = 0.013) and TCLO/VA (beta=-0.18, 95%CI: [-0.33, -0.03], *p* = 0.022, Fig. 4 and Supplementary Table 4). Changes in EMCL were not associated with the presence of COPD (Table 2). The ratio IMCL/EMCL was tentatively associated with higher FEV1/FVC (beta=-0.12, 95%CI: [-0.02, 0.27], *p* = 0.095), as well as with higher TLCO (beta = 0.11, 95%CI: [0.02, 0.20], *p* = 0.021) and TCLO/VA (beta = 0.15, 95%CI: [0.01, 0.29], *p* = 0.031, Fig. 4 and Supplementary Table 4). A higher IMCL/EMCL ratio was associated with a 51% reduced risk for COPD (OR: 0.49, *p* = 0.050, Table 2).

## Discussion

This imaging study demonstrated that (1) myosteatosis, expressed as increased PDFF in paraspinal muscles is associated with obstructive lung disease and impaired lung function parameters, (2) the distribution of fat between different compartments appears particularly relevant: a higher ratio of intramyocellular to extramyocellular lipids was associated with lower risk of obstructive lung disease, suggesting preserved metabolic function.

Several studies have demonstrated muscle alterations in individuals with COPD, with recent evidence highlighting associations between myosteatosis and systemic inflammation, altered muscle bioenergetics, and adverse clinical outcomes [24–26]. Muscle dysfunction represents a well-established extrapulmonary manifestation of COPD, affecting both respiratory and locomotor muscles [4]. Robles et al. have shown that muscle fat infiltration is associated with impaired lower extremity muscle function in COPD patients [27]. In another study by Shields et al., increased intermuscular fat content was correlated with reduced quadriceps strength in COPD patients [28]. Population-based studies examining muscle-respiratory relationships are particularly scarce, as most evidence is derived from clinical cohorts with established diseases [29–31]. The present study extends this research by investigating associations between COPD and muscle fat composition in a general population setting, with a particular focus on IMCL and EMCL evaluation using chemical shift encoded MRI.

The role of skeletal muscle dysfunction in respiratory diseases is well-documented, yet the specific underlying processes remain incompletely understood [32]. Multiple mechanisms may link myosteatosis to obstructive lung disease. Adipose tissue is now recognized as an endocrine organ, and ectopic fat accumulation in various compartments, including skeletal muscle, liver, and visceral adipose tissue, has been linked to metabolic disorders and obesity - conditions known to be associated with impaired respiratory function [33]. Moreover, the pro-inflammatory properties of intermuscular adipose tissue and chronic systemic inflammation in COPD may promote adipogenesis within muscle tissue, potentially contributing to a cycle of increased fat accumulation and inflammatory activity [11, 24, 34]. Further, hypoxia and oxidative stress can impair muscle metabolism and promote lipid accumulation [35], while physical inactivity due to respiratory symptoms may contribute to muscle atrophy and increased fat deposition [36]. While some of these mechanisms, particularly physical inactivity, might suggest altered muscle mass in COPD, we found no significant associations between muscle area and COPD in our study. Although muscle area showed associations with lung volumes and was positively associated with gas exchange capacity — likely reflecting generally larger body sizes —the lack of association with COPD suggests that it is not reduced muscle quantity (e.g., sarcopenia), but rather reduced muscle quality (e.g., myosteatosis) and specifically the pattern of fat distribution, that may be more relevant in obstructive respiratory disease. This is supported by our observation that while higher total myosteatosis showed negative associations, higher IMCL/EMCL ratios showed protective associations with COPD status.

The paraspinal muscles play a critical role in posture, core stability, and respiratory mechanics, with particular involvement during forced respiratory maneuvers [37]. This muscle group contains a high proportion of type I (slow-twitch) oxidative fibers, which characteristically maintain high IMCL levels for aerobic metabolism [38, 39]. In COPD, skeletal muscle may undergo adaptation toward type II (fast-twitch) fibers, which contain less IMCL than type I fibers and exhibit lower fatigue resistance. Of note, the shift towards type II fibers appears to correlate with disease severity in COPD patients [40]. Our findings of protective associations with higher IMCL/EMCL ratios suggests preserved type I fiber characteristics, supported by the concomitant finding of better gas exchange parameters - likely reflecting maintained aerobic muscle metabolism.

These observations of distinct muscle fat compositions may have clinical implications. MRI-derived muscle fat measurements could potentially serve as quantitative markers of muscle alterations in COPD [41]. Exercise-based interventions have shown positive effects on both muscle function and composition in COPD patients [36, 42, 43]. Additionally, novel pharmacotherapeutic agents with mechanisms of action specific to muscle fiber types have been introduced [44]. Following, the potential role of IMCL/EMCL ratio as a marker of muscle adaptation warrants further investigation.

A particular strength of this study is the application of 3 Tesla MRI for muscle assessment. Previous studies predominantly used computed tomography to assess skeletal muscle alterations in COPD, primarily focusing on lower extremity muscle area with limited investigation of qualitative changes of skeletal muscle/myosteatosis [41]. Unlike computed tomography, which provides an indirect assessment of muscle fat content through radiodensity measurements, chemical shift encoded MRI using multi-echo Dixon-based sequences enables detailed tissue characterization without radiation exposure and provides precise and reproducible PDFF measurements corrected for T2* decay and other confounding effects [45–48]. Due to these advantages, PDFF has been established as an imaging biomarker for tissue fat quantification and represents the reference standard for non-invasive fat assessment [49, 50]. Analysis of paraspinal muscles was performed on axial images at the level of the third lumbar vertebra, a standardized anatomical location shown to provide validated surrogate parameters for overall body composition [51]. Recent advances in 3D whole-body MRI-based automated muscle segmentation [52] offer opportunities to extend muscle composition analysis to larger population cohorts and potentially to additional muscle groups relevant to respiratory function, although technical challenges remain for reliably quantifying structures like the diaphragm and intercostal muscles due to respiratory motion and complex anatomy [53].

Some limitations of our study should be considered. The cross-sectional design precludes causal inference regarding the temporal relationship between muscle changes and COPD development. Longitudinal studies are needed to determine whether respiratory impairment drives muscle fat infiltration, whether altered muscle metabolism contributes to respiratory dysfunction, or whether both processes occur simultaneously through shared pathophysiological mechanisms. We consider our results as first signals for a statistical association potentially reflecting a true biological pathway, but our relatively small sample size and the number of associations tested requires replication and validation in larger, external samples. COPD was defined based on spirometric indices without a physician’s diagnosis. However, this approach has been widely applied and accepted in population-based studies, and by using the LLN criterion for FEV1/FVC in line with national as well as ATS/ERS recommendations, we minimized the risk of possibly inadequate classification, especially in older individuals [21, 54, 55]. Finally, direct histological validation was not available in our cohort. However, MRI-based PDFF quantification has demonstrated excellent correlation with histopathological assessment in previous studies [56, 57].

In conclusion, this study demonstrates significant associations between increased paraspinal myosteatosis and spirometrically defined COPD in a population-based sample. The protective associations of higher IMCL/EMCL ratios may reflect preserved type I fiber characteristics, supporting the link between muscle metabolic properties and respiratory function. These observations indicate new opportunities for monitoring disease progression and developing therapeutic strategies in COPD. Larger longitudinal studies are warranted to confirm our findings and evaluate their prognostic implications and potential therapeutic applications.

## Electronic supplementary material

Below is the link to the electronic supplementary material.

Supplementary Material 1

## Data Availability

The datasets analyzed during the current study are not publicly available due to national data protection laws, since the informed consent given by KORA study participants does not cover data posting in public databases. Data are available upon request by means of a project agreement from KORA. Requests should be sent to kora.passt@helmholtz-munich.de and are subject to approval by the KORA Board. Analysis codes are available from the authors upon reasonable request.

## Abbreviations

BMI: Body mass index
COPD: Chronic obstructive pulmonary disease
EMCL: Extramyocellular lipids
FEF_25 − 75_: Forced expiratory flow between 25% and 75% of FVC
FEV1: Forced expiratory volume in 1 s
FRC: Functional residual capacity
FVC: Forced vital capacity
HbA1c: Glycated hemoglobin
HDL: High density lipoprotein
hsCRP: High sensitivity C-reactive protein
IMCL: Intramyocellular lipids
KORA: Cooperative Health Research in the Region of Augsburg
L3: 3rd lumbar vertebra
LDL: Low density lipoprotein
LLN: Lower limit of normal
MRI: Magnetic resonance imaging
MRIvol: MRI derived lung volumes
OR: Odds Ratio
PDFF: Proton density fat fraction
RV: Residual volume
TLC: Total lung capacity
TLCO: Transfer factor of the lung for carbon monoxide
VA: Alveolar volume
