# Supplementary material for: Paraspinal myosteatosis is associated with COPD: a cross-sectional MRI analysis from the population-based KORA cohort
Source: Respir Res. 2025 Jun 14;26:217. doi: 10.1186/s12931-025-03297-4 (PMC12167581; doi:10.1186/s12931-025-03297-4)
Supplement: Supplementary file 1 — Supplementary Material 1 [file 12931_2025_3297_MOESM1_ESM.docx]

Supplementary Material to

**Paraspinal Myosteatosis Is Associated with COPD: A Cross-sectional MRI Analysis from the Population-based KORA Cohort**

Diallo et al.

**Tables**

**Supplementary Table 1**: Demographic and clinical characteristics of individuals excluded from the final analysis, and individuals included in the final analysis

|  | Excluded | Included | p-value |
| --- | --- | --- | --- |
|  | N = 186 | N = 214 |  |
| **Demographics** |  | |  |
| Age, years | 53.9 ± 11.6 | 58.5 ± 5.8 | <0.001 |
| Men | 110 (59.1%) | 120 (56.1%) | 0.561 |
| **Body composition** |  | |  |
| Height, cm | 172.3 ± 9.4 | 171.0 ± 10.0 | 0.201 |
| Weight, kg | 82.1 ± 17.3 | 83.8 ± 15.9 | 0.325 |
| BMI, kg/m2 | 27.6 ± 5.1 | 28.6 ± 4.7 | 0.042 |
| BMI≥30 kg/m2 | 53 (28.5%) | 69 (32.2%) | 0.504 |
| Waist circumference, cm | 96.7 ± 15.2 | 100.2 ± 13.4 | 0.015 |
| Body Surface Area, m2 | 1.9 ± 0.2 | 2.0 ± 0.2 | 0.723 |
| **Lipid Profile** |  |  |  |
| Total Cholesterol, mg/dL | 212.7 ± 34.6 | 222.4 ± 37.2 | 0.008 |
| Triglycerides, mg/dL (median[Q1, Q3]) | 104.0 [72.1, 149.0] | 113.0 [83.2, 163.6] | 0.054 |
| HDL, mg/dL | 61.6 ± 17.7 | 62.2 ± 17.7 | 0.733 |
| LDL, mg/dL | 135.8 ± 30.8 | 142.9 ± 34.4 | 0.034 |
| Lipid lowering medication | 16 (8.6%) | 27 (12.6%) | 0.266 |
| **Glycemia** |  |  |  |
| Status |  |  | 0.184 |
| normogylcemic | 121 (65.1%) | 121 (56.5%) |  |
| prediabetes | 43 (23.1%) | 60 (28.0%) |  |
| diabetes | 21 (11.3%) | 33 (15.4%) |  |
| Fasting glucose, mg/dL | 101.9 ± 23.5 | 106.3 ± 21.7 | 0.054 |
| HbA1c, % | 5.5 ± 0.8 | 5.6 ± 0.6 | 0.044 |
| Glucose lowering medication | 13 (7.0%) | 19 (8.9%) | 0.621 |
| **Blood pressure** |  | |  |
| Systolic blood pressure, mmHg | 119.0 ± 17.5 | 122.1 ± 16.0 | 0.061 |
| Diastolic blood pressure, mmHg | 73.6 ± 10.1 | 76.8 ± 9.7 | 0.002 |
| Hypertension | 51 (27.4%) | 85 (39.7%) | 0.014 |
| Antihypertensive medication | 38 (20.4%) | 64 (29.9%) | 0.043 |
| **Lifestyle factors** |  | |  |
| Physical activity |  | | 0.910 |
| regularly 2h/w | 55 (29.6%) | 59 (27.6%) |  |
| regularly 1h/w | 54 (29.0%) | 69 (32.2%) |  |
| sporadically | 26 (14.0%) | 31 (14.5%) |  |
| inactive | 50 (26.9%) | 55 (25.7%) |  |
| Smoking |  |  | 0.440 |
| never-smoker | 63 (33.9%) | 82 (38.3%) |  |
| ex-smoker | 87 (46.8%) | 87 (40.7%) |  |
| smoker | 35 (18.8%) | 45 (21.0%) |  |
| **Other laboratory markers** |  | |  |
| Vitamin D (Calciferol), ng/ml | 23.3 ± 12.2 | 23.8 ± 11.3 | 0.691 |
| hsCRP, mg/L | 1.1 [0.6, 2.3] | 1.2 [0.7, 2.8] | 0.219 |
| **Lung volume, MRI** |  | |  |
| total, L | 4.0 ± 1.1 | 4.0 ± 1.1 | 0.661 |
| left, L | 1.8 ± 0.5 | 1.8 ± 0.6 | 0.631 |
| right, L | 2.2 ± 0.6 | 2.2 ± 0.6 | 0.697 |

Values are presented as arithmetic means and standard deviations (or otherwise indicated) with p-values from t-test (or Mann-Whitney-U test), or counts and percentages with p-values from Χ^2^-test.

**Abbreviations:** BMI: body mass index; HbA1c: glycated hemoglobin; HDL: high density lipoprotein; hsCRP: high sensitivity C-reactive protein; LDL: low density lipoprotein; MRI: magnetic resonance imaging.

**Supplementary Table 2:** Association between skeletal muscle parameters and outcomes of lung volumes.

|  | Outcome | RV | | | FRC | | | VA | | | TLC | | | MRI Lung Volume | | |
| --- | --- | --- | --- | --- | --- | --- | --- | --- | --- | --- | --- | --- | --- | --- | --- | --- |
| Exposure | Adjustment | beta | 95%-CI | p | beta | 95%-CI | p | beta | 95%-CI | p | beta | 95%-CI | p | beta | 95%-CI | p |
| Area | Age+sex | -0.01 | [-0.16, 0.14] | 0.892 | -0.12 | [-0.27, 0.03] | 0.112 | 0.05 | [-0.07, 0.16] | 0.417 | 0.06 | [-0.05, 0.17] | 0.296 | -0.07 | [-0.22, 0.08] | 0.385 |
|  | Full | 0.1 | [-0.05, 0.26] | 0.172 | 0.07 | [-0.06, 0.20] | 0.282 | 0.16 | [0.05, 0.27] | 0.003 | 0.17 | [0.06, 0.27] | 0.003 | 0.01 | [-0.14, 0.17] | 0.853 |
|  | Full+hsCRP | 0.13 | [-0.03, 0.28] | 0.104 | 0.08 | [-0.05, 0.21] | 0.230 | 0.16 | [0.05, 0.27] | 0.005 | 0.16 | [0.05, 0.27] | 0.004 | 0.01 | [-0.15, 0.17] | 0.877 |
| PDFF | Age+sex | 0 | [-0.13, 0.13] | 0.969 | -0.16 | [-0.29, -0.03] | 0.015 | -0.11 | [-0.20, -0.01] | 0.029 | -0.1 | [-0.19, -0.00] | 0.044 | -0.03 | [-0.16, 0.10] | 0.682 |
|  | Full | 0.1 | [-0.04, 0.23] | 0.153 | 0 | [-0.12, 0.11] | 0.979 | -0.02 | [-0.12, 0.08] | 0.709 | -0.02 | [-0.12, 0.08] | 0.717 | 0.05 | [-0.09, 0.18] | 0.518 |
|  | Full+hsCRP | 0.09 | [-0.05, 0.22] | 0.219 | 0 | [-0.12, 0.11] | 0.965 | -0.02 | [-0.12, 0.08] | 0.717 | -0.02 | [-0.12, 0.08] | 0.724 | 0.05 | [-0.09, 0.19] | 0.517 |
| IMCL | Age+sex | -0.03 | [-0.16, 0.10] | 0.645 | -0.12 | [-0.24, 0.01] | 0.062 | -0.06 | [-0.15, 0.03] | 0.197 | -0.05 | [-0.14, 0.04] | 0.247 | -0.05 | [-0.18, 0.08] | 0.448 |
|  | Full | 0.03 | [-0.10, 0.16] | 0.638 | -0.02 | [-0.13, 0.09] | 0.709 | 0.01 | [-0.08, 0.10] | 0.835 | 0.01 | [-0.08, 0.10] | 0.823 | -0.00 | [-0.13, 0.13] | 0.990 |
|  | Full+hsCRP | 0.03 | [-0.10, 0.16] | 0.639 | -0.01 | [-0.12, 0.10] | 0.797 | -0.00 | [-0.10, 0.09] | 0.963 | -0.00 | [-0.10, 0.09] | 0.973 | -0.02 | [-0.15, 0.12] | 0.809 |
| EMCL | Age+sex | -0.05 | [-0.18, 0.08] | 0.435 | -0.17 | [-0.30, -0.05] | 0.007 | -0.13 | [-0.22, -0.03] | 0.008 | -0.12 | [-0.21, -0.03] | 0.012 | -0.09 | [-0.22, 0.04] | 0.197 |
|  | Full | 0.03 | [-0.10, 0.17] | 0.607 | -0.04 | [-0.15, 0.07] | 0.476 | -0.05 | [-0.14, 0.04] | 0.285 | -0.05 | [-0.14, 0.04] | 0.291 | -0.02 | [-0.16, 0.11] | 0.745 |
|  | Full+hsCRP | 0.03 | [-0.11, 0.16] | 0.691 | -0.04 | [-0.15, 0.07] | 0.499 | -0.05 | [-0.15, 0.04] | 0.261 | -0.05 | [-0.15, 0.04] | 0.266 | -0.02 | [-0.16, 0.11] | 0.731 |
| IMCL/EMCL | Age+sex | -0.01 | [-0.14, 0.11] | 0.815 | 0.08 | [-0.04, 0.20] | 0.179 | 0.08 | [-0.01, 0.16] | 0.087 | 0.07 | [-0.01, 0.16] | 0.103 | 0.05 | [-0.08, 0.17] | 0.465 |
|  | Full | -0.08 | [-0.19, 0.04] | 0.204 | -0.02 | [-0.12, 0.08] | 0.706 | 0.03 | [-0.05, 0.11] | 0.471 | 0.03 | [-0.05, 0.12] | 0.468 | 0.01 | [-0.12, 0.13] | 0.905 |
|  | Full+hsCRP | -0.08 | [-0.20, 0.04] | 0.214 | -0.02 | [-0.13, 0.08] | 0.628 | 0.03 | [-0.06, 0.12] | 0.489 | 0.03 | [-0.06, 0.12] | 0.487 | 0 | [-0.13, 0.12] | 0.949 |

Results from a linear regression model with exposure muscle parameters, as derived by MRI, and outcome lung volumes. Full adjustment: Adjusted for age, sex, smoking, diabetes, physical activity (yes/no), and BMI. All exposures and outcomes were standardized before modelling. Results for the full model are presented in Figure 3 in the main manuscript.

**Abbreviations:** BMI: body mass index; EMCL: extramyocellular lipids; FRC: functional residual capacity; IMCL: intramyocellular lipids; MRI: magnetic resonance imaging; PDFF: proton density fat fraction; RV: residual volume; TLC: total lung capacity; VA: alveolar volume.

**Supplementary Table 3:** Association between muscle parameters and outcomes of lung obstruction.

|  | | Outcome | FVC | | | FEV1/FVC | | | FEF_25-75_ | | |
| --- | --- | --- | --- | --- | --- | --- | --- | --- | --- | --- | --- |
| Exposure | | Adjustment | beta | 95%-CI | p | beta | 95%-CI | p | beta | 95%-CI | p |
| Area | | Age+sex | 0.03 | [-0.08, 0.14] | 0.535 | 0.13 | [-0.04, 0.31] | 0.140 | 0.06 | [-0.11, 0.23] | 0.483 |
|  |  | Full | 0.15 | [0.04, 0.26] | 0.008 | 0.05 | [-0.13, 0.24] | 0.567 | 0.05 | [-0.13, 0.23] | 0.597 |
|  |  | Full+hsCRP | 0.14 | [0.02, 0.25] | 0.017 | 0.03 | [-0.16, 0.22] | 0.744 | 0.03 | [-0.15, 0.21] | 0.732 |
| PDFF | | Age+sex | -0.10 | [-0.20, -0.00] | 0.041 | -0.09 | [-0.24, 0.06] | 0.249 | -0.12 | [-0.27, 0.03] | 0.116 |
|  |  | Full | -0.02 | [-0.12, 0.08] | 0.654 | -0.17 | [-0.33, -0.01] | 0.033 | -0.13 | [-0.29, 0.02] | 0.092 |
|  |  | Full+hsCRP | -0.02 | [-0.12, 0.08] | 0.666 | -0.15 | [-0.31, 0.01] | 0.070 | -0.12 | [-0.28, 0.04] | 0.155 |
| IMCL | Age+sex | -0.04 | [-0.13, 0.05] | 0.351 | 0.04 | [-0.10, 0.19] | 0.565 | -0.02 | [-0.17, 0.12] | 0.742 |  |
|  | Full | 0.02 | [-0.07, 0.11] | 0.597 | 0 | [-0.15, 0.15] | 0.981 | -0.02 | [-0.17, 0.13] | 0.826 |  |
|  | Full+hsCRP | 0 | [-0.09, 0.10] | 0.939 | 0.02 | [-0.14, 0.18] | 0.802 | 0 | [-0.15, 0.16] | 0.982 |  |
| EMCL | Age+sex | -0.11 | [-0.20, -0.02] | 0.021 | -0.01 | [-0.16, 0.15] | 0.938 | -0.07 | [-0.21, 0.08] | 0.378 |  |
|  | Full | -0.04 | [-0.13, 0.06] | 0.435 | -0.07 | [-0.23, 0.08] | 0.365 | -0.08 | [-0.23, 0.08] | 0.328 |  |
|  | Full+hsCRP | -0.04 | [-0.14, 0.05] | 0.366 | -0.05 | [-0.21, 0.11] | 0.533 | -0.06 | [-0.21, 0.10] | 0.456 |  |
| IMCL/EMCL | | Age+sex | 0.07 | [-0.02, 0.16] | 0.118 | 0.09 | [-0.05, 0.24] | 0.205 | 0.08 | [-0.06, 0.22] | 0.244 |
|  |  | Full | 0.03 | [-0.05, 0.12] | 0.475 | 0.14 | [0.00, 0.28] | 0.050 | 0.1 | [-0.04, 0.24] | 0.154 |
|  |  | Full+hsCRP | 0.03 | [-0.05, 0.12] | 0.463 | 0.12 | [-0.02, 0.27] | 0.095 | 0.09 | [-0.05, 0.23] | 0.217 |

Results from a linear regression model with exposure muscle parameters, as derived by MRI, and outcome parameters of

lung obstruction. Full adjustment: Adjusted for age, sex, smoking, diabetes, physical activity (yes/no), and BMI.

All exposures and outcomes were standardized before modelling. Results for the full model are presented in Figure 3

in the main manuscript.

**Abbreviations:** BMI: body mass index; EMCL: extramyocellular lipids; FEF_25-75:_ forced expiratory flow between 25% and 75% of FVC;

FEV_1_/FVC: Tiffenau-Index; FVC: forced vital capacity; IMCL: intramyocellular lipids; MRI: magnetic resonance imaging;

PDFF: proton density fat fraction.

**Supplementary Table 4:** Association between muscle parameters and outcomes of gas exchange.

|  | | Outcome | | TLCO | | | | | TLCO/VA | | | | |
| --- | --- | --- | --- | --- | --- | --- | --- | --- | --- | --- | --- | --- | --- |
|  | | Adjustment | | beta | | CI | | p | beta | | CI | | p |
| Area (mm^2^) | | Age+sex | | 0.17 | | [0.05, 0.28] | | 0.005 | 0.2 | | [0.02, 0.38] | | 0.032 |
|  |  | Full | | 0.2 | | [0.09, 0.32] | | 0.001 | 0.08 | | [-0.10, 0.25] | | 0.380 |
|  |  | Full+hsCRP | | 0.2 | | [0.08, 0.32] | | 0.001 | 0.08 | | [-0.09, 0.26] | | 0.353 |
| PDFF (%) | | Age+sex | | -0.13 | | [-0.23, -0.03] | | 0.011 | -0.08 | | [-0.24, 0.08] | | 0.337 |
|  |  | Full | | -0.12 | | [-0.22, -0.01] | | 0.030 | -0.19 | | [-0.35, -0.04] | | 0.014 |
|  |  | Full+hsCRP | | -0.11 | | [-0.22, -0.00] | | 0.043 | -0.19 | | [-0.35, -0.04] | | 0.016 |
| IMCL (%) | | Age+sex | -0.09 | [-0.19, 0.00] | | 0.054 | | -0.08 | [-0.23, 0.07] | | 0.289 | |  |
|  |  | Full | -0.07 | [-0.16, 0.03] | | 0.177 | | -0.15 | [-0.29, -0.00] | | 0.048 | |  |
|  |  | Full+hsCRP | -0.07 | [-0.17, 0.03] | | 0.173 | | -0.14 | [-0.29, 0.01] | | 0.074 | |  |
| EMCL (%) | | Age+sex | -0.15 | [-0.24, -0.05] | | 0.003 | | -0.08 | [-0.24, 0.07] | | 0.290 | |  |
|  |  | Full | -0.13 | [-0.23, -0.03] | | 0.010 | | -0.18 | [-0.33, -0.03] | | 0.018 | |  |
|  |  | Full+hsCRP | -0.13 | [-0.23, -0.03] | | 0.013 | | -0.18 | [-0.33, -0.03] | | 0.022 | |  |
| IMCL/EMCL | | Age+sex | | 0.11 | | [0.02, 0.21] | | 0.014 | 0.08 | | [-0.06, 0.23] | | 0.249 |
|  |  | Full | | 0.11 | | [0.02, 0.20] | | 0.017 | 0.15 | | [0.01, 0.28] | | 0.031 |
|  |  | Full+hsCRP | | 0.11 | | [0.02, 0.20] | | 0.021 | 0.15 | | [0.01, 0.29] | | 0.031 |

Results from a linear regression model with exposure muscle parameters, as derived by MRI, and outcome parameters of

gas exchange. Full adjustment: Adjusted for age, sex, smoking, diabetes, physical activity (yes/no), and BMI.

All exposures and outcomes were standardized before modelling. Results for the full model are presented in Figure 3

in the main manuscript.

**Abbreviations:** BMI: body mass index; EMCL: extramyocellular lipids; IMCL: intramyocellular lipids; MRI: magnetic resonance imaging;

PDFF: proton density fat fraction; TLCO: transfer factor of the lung for carbon monoxide; VA: alveolar volume.

**FIGURES**


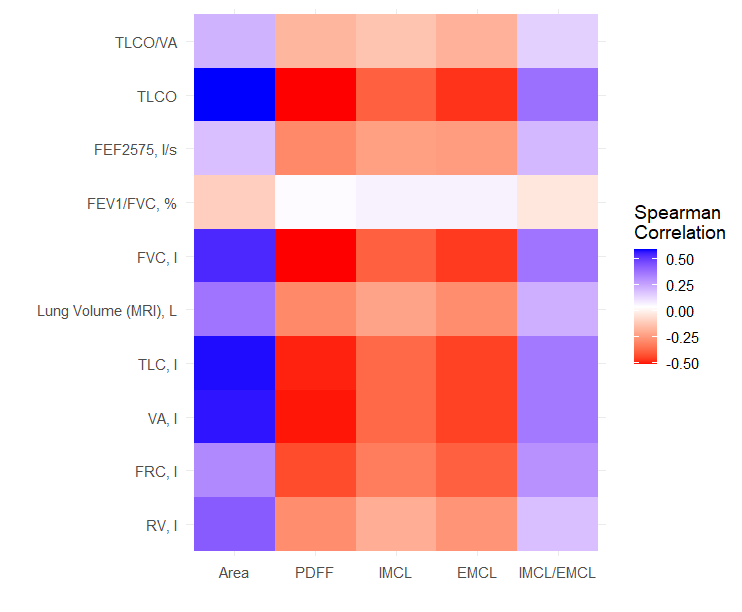


**Supplementary Figure 1:** Univariate correlations between muscle PDFF and pulmonary function parameters

**Abbreviations:** EMCL: extramyocellular lipids; FEF_25-75:_ forced expiratory flow between 25% and 75% of FVC; FEV_1_/FVC: Tiffenau-Index; FRC: functional residual capacity; FVC: forced vital capacity; IMCL: intramyocellular lipids; MRl: magnetic resonance imaging; RV: residual volume; TLC: total lung capacity; TLCO: transfer factor of the lung for carbon monoxide; VA: alveolar volume.
